# Linking Cancer Metabolic Dysfunction and Genetic Instability through the Lens of Iron Metabolism

**DOI:** 10.3390/cancers11081077

**Published:** 2019-07-30

**Authors:** Michael S. Petronek, Douglas R. Spitz, Garry R. Buettner, Bryan G. Allen

**Affiliations:** Free Radical and Radiation Biology Program, Department of Radiation Oncology, Free Radical Metabolism and Imaging Program, Holden Comprehensive Cancer Center, The University of Iowa, Iowa City, IA 52242, USA

**Keywords:** iron metabolism, cancer, mitochondrial iron, mitochondria, genetic theory of cancer, genetic instability, metabolism, labile iron pool, ferritin, transferrin receptors

## Abstract

Iron (Fe) is an essential element that plays a fundamental role in a wide range of cellular functions, including cellular proliferation, DNA synthesis, as well as DNA damage and repair. Because of these connections, iron has been strongly implicated in cancer development. Cancer cells frequently have changes in the expression of iron regulatory proteins. For example, cancer cells frequently upregulate transferrin (increasing uptake of iron) and down regulate ferroportin (decreasing efflux of intracellular iron). These changes increase the steady-state level of intracellular redox active iron, known as the labile iron pool (LIP). The LIP typically contains approximately 2% intracellular iron, which primarily exists as ferrous iron (Fe^2+^). The LIP can readily contribute to oxidative distress within the cell through Fe^2+^-dioxygen and Fenton chemistries, generating the highly reactive hydroxyl radical (HO^•^). Due to the reactive nature of the LIP, it can contribute to increased DNA damage. Mitochondrial dysfunction in cancer cells results in increased steady-state levels of hydrogen peroxide and superoxide along with other downstream reactive oxygen species. The increased presence of H_2_O_2_ and O_2_^•−^ can increase the LIP, contributing to increased mitochondrial uptake of iron as well as genetic instability. Thus, iron metabolism and labile iron pools may play a central role connecting the genetic mutational theories of cancer to the metabolic theories of cancer.

## 1. Introduction

While not recognized as a traditional hallmark of cancer, increased iron availability is an integral feature of neoplastic disease. Iron is an essential element that is tightly regulated at multiple levels in normal cells. It is imperative that appropriate levels of iron are available for key cellular functions including aerobic metabolism, repair of DNA damage, and cell cycle progression [1,2,3,4,5]. Alternatively, while iron availability is essential, an overabundance of iron can be detrimental. Increased cellular iron has the potential to increase oxidative distress. This can be through its reaction with dioxygen [6], as well as classical Fenton chemistry leading to the production of the highly reactive hydroxyl-radical from H_2_O_2_ (HO^•^) [7], leading to organic hydroperoxides, organic radicals, and aldehydic by-products of lipid and amino acid oxidation. Alterations in iron metabolism have been linked to various diseases, including Alzheimer’s disease, Parkinson’s disease, Friedreich Ataxia, chronic kidney disease, and iron deficient anemia [8,9,10].

Iron is readily able to participate in one-electron reactions, cycling between Fe^3+^ and Fe^2+^ oxidation states [11]. Intracellular iron is present in heme groups, Fe-S clusters, as well as free, redox active “labile” iron. Heme proteins are important not only for the transfer of oxygen, most famously, hemoglobin [12], but also as agents for the transfer of electrons in both 1-e^−^ and 2-e^−^ oxidation-reduction reactions. Proteins with Fe-S clusters also readily undergo redox reactions and therefore primarily function as electron transfer agents, e.g., electron transport chain complexes, and a wide variety of oxidoreductases [13]; they can also facilitate other reactions, such as the isomerization of citrate by aconitase. Proteins containing Fe-S clusters can have [2Fe-2S], [4Fe-4S], or [3Fe-4S] cluster organization with either Fe^3+^ (oxidized) or Fe^3+^/Fe^2+^ (reduced) centers to facilitate electron transfer.

The iron in the labile iron pool (LIP) exists primarily in the ferrous, Fe^2+^, oxidation state [14]. The ferrous labile iron pool is only a small portion of the total iron in cells (≤2%), but this iron is the central hub of the iron metabolic network [4,5]. While it provides the necessary iron reserve for incorporation into proteins, it can also contribute to oxidative distress in cells by facilitating oxidations via reactions with dioxygen, Fe^2+^ + O_2_ → ‘Fe^2+^-O_2_’ [6]. These ‘Fe^2+^-O_2_’ complexes will lead to the initiation of the oxidation of a wide range of biomolecules, similar to classical Fenton chemistry [4,6]. Ferrous iron can react with H_2_O_2_ to form the hydroxyl radical, HO^•^ (Equation (1)), i.e., the Fenton reaction [4,7,11,15,16].

(1) $${\mathrm{Fe}}^{2+}+H_{2}O_{2}\to {\mathrm{Fe}}^{3+}+{\mathrm{OH}}^{-}+{\mathrm{HO}}^{•}$$

**Figure 1:**
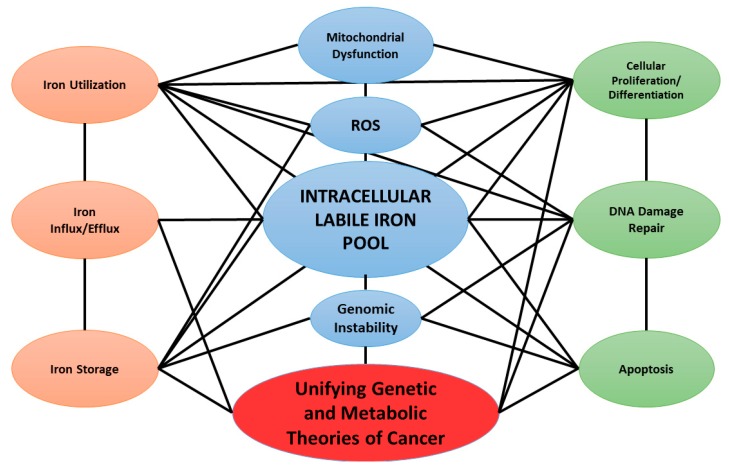
Simplified model of the network for intracellular iron regulation. This model unifies the genetic and metabolic theories of cancer where the intracellular labile iron pool (LIP) acts as a central hub in the genesis of cancer thereby linking iron metabolism to traditional hallmarks of cancer.

Increasing evidence suggests that the altered regulation of iron metabolism plays an essential role in cancer initiation and progression [17,18,19]. Several cancer cell types have demonstrated increases in their LIP relative to adjacent normal tissues [20,21]. Schoenfeld et al. have shown that both lung and brain cancer cells may have more than a two-fold increase in LIP relative to normal human cells [21]. This review focuses on the genomic alterations of iron metabolism in cancer as well as other pathways for increasing the LIP, such as metabolic perturbations. Iron may provide a mechanistic link between metabolic and genetic theories of cancer (Figure 1).

## 2. Overview of Intracellular Iron Metabolism

### 2.1. Iron Uptake, Storage, and Homeostasis

Upon capture of iron from the digestive track, it is escorted through the blood stream by transferrin, a protein that binds two ferric iron (Fe^3+^) ions. Cells in need of iron express transferrin receptors (TfR) on the extracellular surface of their plasma membrane. Di-ferric transferrin (Tf) then binds to a TfR, which initiates internalization by endocytosis. Once endosomally internalized, the low pH of the vacuole allows for the dissociation of the two iron ions from the di-ferric Tf-TfR complex. Upon release, this ferric iron is reduced to ferrous iron by ferrireductases and then exported from the vacuole by divalent metal transporter–1 (DMT1). Once in the cytosol, Fe^2+^ can be: stored in ferritin (Ft); utilized to form Fe-S or heme-containing proteins; or contribute to the labile iron pool [15,19,22].

The size of the LIP, i.e., the level of iron in the LIP, is regulated by binding and storing excess iron in ferritin (Ft) or by exporting excess iron from the cell through ferroportin (FPN-1). Ft binds and stores iron in the ferric state [23]. The heavy chain of ferritin (Ft-H) has ferroxidase activity facilitating the conversion Fe^2+^ to Fe^3+^ iron and then storage within the protein [24]. In order for iron to be exported or stored, it must be transported to either Ft or FPN-1 by the chaperone protein, Poly r(C)-Binding Protein 1/2 (PCBP1/2) [25,26,27,28].

The LIP is used as a feedback mechanism to regulate Ft, TfR, and FPN-1 expression [29]. The mRNAs for these proteins contain hairpin loop structures that act as iron response elements (IREs). Under low iron conditions, iron responsive proteins 1/2 (IRP1/2) bind to the IREs at the 3’ untranslated region (UTR) of TfR mRNA stabilizing it for translation and facilitating intracellular iron uptake. Similarly, IRP1/2 bind to IREs at the 5’ UTR of Ft and FPN-1 to block its translation and prohibit intracellular iron storage and export. When the LIP increases, IRP1/2 dissociates from the respective IREs allowing for the degradation of the TfR mRNA transcript and translation of Ft and FPN-1 mRNAs facilitating iron storage and export [29].

IRP1/2 are key iron regulators for the maintenance of LIP homeostasis. IRP1 is cytosolic aconitase, an enzyme containing a [4Fe-4S] cluster. When the concentration of intracellular iron is low, there is insufficient iron for Fe-S biogenesis leaving an incomplete [3Fe-4S] cluster [22]. The enzymatic activity of aconitase is lost and this protein then initiates its IRP activity, as IRP1. When the protein contains the [3Fe-4S] cluster it can bind to IREs. While IRP1 is directly regulated by the intracellular iron concentration, IRP2 is regulated via degradation [22,29]. Under high iron concentrations, IRP2 is ubiquitinated by the SKP1-CUL1-FBXL5 E3 ubiquitin ligase complex, which is consequently responsive to intracellular iron concentrations [30]. Thus, the size of the LIP is sensed, and actions are initiated to either decrease or increase the level of iron in the LIP.

### 2.2. Mitochondrial Iron Metabolism

Iron metabolism is heavily dependent on mitochondrial function. While mitochondria are known as the driver of ATP production through oxidative phosphorylation, they also house the necessary machinery for iron utilization. The machinery for both heme synthesis and iron sulfur cluster biogenesis are present in mitochondria. Thus, the incorporation of iron into many important proteins occurs in mitochondria.

Despite the poorly understood mechanisms driving iron trafficking from the LIP to mitochondria, the mechanism for the influx of iron across the inner mitochondrial membrane is well-established. Iron is brought into mitochondria across the inner mitochondrial membrane through the iron transporters, mitoferrin-1 and -2 (MFRN-1/2) [31,32]. MFRN-1/2 are stabilized on the inner mitochondrial membrane by ABCB10 (ATP-binding Cassette Sub-family B Member 10). Reduced expression of either MFRN or ABCB10 results in mitochondrial iron depletion [32,33,34,35]. Once iron is brought into the mitochondria, it is utilized in three major metabolic pathways: heme synthesis, iron sulfur cluster biogenesis, and iron storage.

### 2.3. Heme Synthesis

Heme synthesis occurs in the mitochondria of all cells, but is especially relevant in hepatocytes and erythroid cells [36]. Heme synthesis is an 8-step process occurring in both the mitochondria and the cytosol [36,37]. The first step of heme synthesis is catalyzed by δ-aminolevulinic acid synthase (ALAS) in the mitochondrial matrix. This is a condensation reaction between glycine and succinyl CoA producing δ-aminolevulinic acid (ALA). ALAS has two isoforms: ubiquitously expressed ALAS1, and erythroid specific ALAS2. The subsequent 4 steps occur in the cytosol with two ALA molecules being converted to porphobilinogen (PBG). PBG undergoes two subsequent enzymatic steps in the cytosol that results in the production of coproporphyrinogen III (CoPIII). CoPIII is converted to protoporphyrinogen IX (PPIX) and then PPIX has one atom of Fe^2+^ inserted into it by ferrochelatase (FECH) in the mitochondrial inner membrane space [36,37,38]. The rate-limiting step(s) of heme synthesis is the acquisition of iron from Tf in erythroid cells and the formation of ALA in non-erythroid cells [36,37].

### 2.4. Iron Sulfur Cluster Biogenesis

Along with heme synthesis, biogenesis of iron sulfur clusters (Fe-S) is the other essential iron metabolic function of mitochondria. There are a multitude of Fe-S proteins that are essential in a wide range of biochemical processes. The most prominent Fe-S proteins are in the electron transport system (ETS). Complex I in the ETS contains nine Fe-S clusters, complex II contains three Fe-S clusters, and complex III contains one Fe-S cluster [2,39,40]. While Fe-S clusters are important for electron transport, they also have important enzymatic activity. For example, aconitase contains a [4Fe-4S] cluster that is necessary for its enzyme activity [22]. Additional roles of Fe-S proteins include DNA replication and repair mediated by DNA polymerases and glycosylases [41].

The process of mammalian Fe-S biogenesis is quite complex but can be broken down into two basic steps: cluster assembly, and incorporation into apo-proteins. While a basic overview is provided below, please refer to Braymer and Lill [42] for a detailed review. Initial cluster formation occurs on the Fe-S cluster assembly enzyme (ISCU), which is the main scaffolding protein [43]. The cysteine desulfurase, NSF1, dimerizes and binds to an ISCU monomer, which releases a sulfur through a cysteine to alanine conversion [37,42,43,44]. The LYR motif-containing protein 4 (LYRM4) is required for NSF1 function as it contains the cysteine residue from which sulfur is removed for cluster formation [43,45]. Frataxin, most known for its role in Fredrich’s Ataxia, plays an important role in this process as it stores and delivers iron to ISCU to allow for Fe-S cluster formation [43,45,46]. The disruption of the complex and release of the Fe-S cluster is facilitated by the chaperones HSPA9/HSC20 [43]. Once released, the loading of Fe-S clusters onto recipient proteins is still poorly understood. It has been suggested that ABCB7 (ATP-binding Cassette Sub-family B Member 7) plays a role in mitochondrial Fe-S cluster export. While it is well-known that the Fe-S biogenesis machinery is necessary, it is still unclear as to whether Fe-S clusters proteins are assembled in mitochondria and exported to the cytosol or vice versa as there are 11 known cytosolic Fe-S assembly factors (CIAs) [43,44].

Three Fe-S proteins localized to the outer mitochondrial membrane are involved in Fe-S biogenesis, CISD1 (mitoNEET), nutrient-deprivation autophagy factor-1 (NAF-1/CISD2), and mitochondrial inner NEET protein (MiNT/CISD3) [47]. These proteins have a reputed role in trafficking Fe-S clusters from the mitochondria to the cytosol. Each of these proteins donate their two [2Fe-2S] clusters to apo-proteins for completion of their Fe-S formation [48,49,50]. MitoNEET has been implicated in iron metabolism by transferring its [2Fe-2S] clusters to: aconitase/IRP1 [49]; the CIA anamorsin/NDOR1 complex [50,51]; redox regulation via interaction with glutathione disulfide reductase (GR) [52]; and metabolism and insulin regulation with glutamate dehydrogenase 1 [53]. Along with mitoNEET, NAF-1 interacts with anamorsin/NDOR1 implicating it in iron metabolism [51]. Interestingly, NAF-1 and mitoNeet interact with one another, which may indicate a role in trafficking Fe-S clusters from mitochondria to the cytosol [54]. The heterodimeric, inner mitochondrial MiNT interacts with mitochondrial ferredoxins (FDX1/2) [55].

### 2.5. Mitochondrial Iron Storage

Iron can also be stored within the inner mitochondrial membrane in mitochondrial ferritin (MtFt), which is structurally similar to cytosolic Ft-H. Being structurally similar to cytosolic Ft-H, iron in MtFt may also be labilized by mitochondrial oxidative stress. MtFt differs from cytosolic Ft as its mRNA does not have the traditional IREs for iron regulation through the IRP/IRE mechanism [56]. MtFt is an intronless gene with a minimal promoter region. MtFt is transcriptionally regulated by SP1, CREB and YY1 as positive regulators and GATA2, FoxA1 and C/EBPβ as negative regulators of MtFt expression [57]. Although there is limited understanding on the role of MtFt in cancer, its simplistic, transcriptional regulation suggests it may be susceptible to carcinogenic alterations.

## 3. Iron and Cancer

Alterations in iron metabolism is a common theme in cancer, with neoplastic cells appearing to have an iron-addiction phenotype [17,18,19,58,59,60,61]. An epidemiological study of 309,443 Taiwanese adults with no history of cancer showed that high serum iron, at the time of recruitment, (≥120 μg dL^−1^), had a 25% increase in cancer incidence risk (all cancers; HR = 1.25; 95% CI 1.16–1.35) and a 39% increase in cancer mortality risk (all cancers; HR 1.39; 95% CI 1.23–1.57) upon long-term follow-up [62]. There was a dose-dependent relationship with a 4% increase in cancer incidence for every 10 μg dL^−1^ above 80 μg dL^−1^ serum iron concentration. Patients with hereditary haemochromatosis (HH), a genetic disease in which individuals are prone to iron overload in tissues (liver in particular) have a relative risk of 1.8 for both hepatocellular carcinoma (95% CI 1.1–2.9) and non-hepatic tumors (95% CI 0.8–4). After adjustment for alcohol abuse, smoking, and family history, HH patients have a relative risk of 1.9 for all cancers (95% CI 1.1–3.1) [63].

A recent retrospective study of breast cancer showed a link between cancer progression and iron metabolism by identifying an iron regulatory gene signature (IRGS) that was predictive for increased tumor progression [64]. Microarray analysis of 674 breast tumors identified 16 genes in the iron metabolic network that were predictive for distant metastasis-free survival (DMFS). When compared to traditional breast cancer prognostic indicators (hormone receptor, lymph nodes, tumor size, and patient age), IRGS was shown to add prognostic value to the prediction of DMFS. These studies illuminate the potentially key roles that iron metabolism play in carcinogenesis and cancer progression.

### 3.1. Iron and Traditional Hallmarks of Cancer

Iron metabolism has been implicated in cancer initiation and progression. Increased iron availability is essential for DNA synthesis and cellular proliferation. Both the constitutive ribonucleotide reductase (RNR) M2 subunit and the p53-inducible RNR M2 subunit contain a critical di-iron site for catalytic activity in the conversion of ribonucleotides to deoxyribonucleotides for DNA synthesis [19,65,66]. Similarly, iron depletion induces cellular G1-arrest [67]. The impact of iron on cell cycle progression extends beyond RNR as cyclinD/cyclin-dependent kinase 4 (CDK4) are degraded and protein levels are reduced by iron depletion [68,69]. Iron depletion has also been shown to increase both p53 and p21 expression, leading to increased CDK4 inhibition and cell cycle arrest [70,71]. Conversely, iron overload increases cyclin D1 expression and cell proliferation in mouse hepatocytes [1]. These observations suggest that increased intracellular iron supports increased cellular proliferation as p53,RNR, cyclinD1/CDK4, and p21 are all implicated in carcinogenesis [3,72,73].

Iron is also essential for DNA metabolism; thus, iron is essential for the maintenance of genomic stability. DNA polymerases I, II, and III have conserved cysteine residues in their active site that allow for the formation of [4Fe-4S] clusters [74]. These [4Fe-4S] clusters are necessary for the catalytic activity of DNA polymerase. Several DNA helicases and glycosylases possess a conserved Fe-S cluster at their N-terminus, which is essential for enzymatic activity [75]. A few examples of such enzymes are: XPD, an SF2-DNA helicase involved in nucleotide excision repair [76]; CHLR1, a helicase that facilitates sister chromatid adhesion to promote genetic stability [77]; and NTHL1, a DNA glycosylase involved in base excision repair [74,75]. A large majority of enzymes responsible for genomic stability are dependent on the catalytic activity Fe-S clusters, thoroughly reviewed by Puig, et al. [74] and Zhang [75].

Iron metabolism also has been shown to have an impact on cell death. Numerous studies have shown that an imbalance of iron is capable of inducing apoptosis [78]. Iron overloaded bone marrow stem cells have been shown to increase expression of pro-apoptotic proteins BAX and cleaved caspase-3, as well as decreased expression of the anti-apoptotic protein Bcl-2. These cells also exhibited an increase in necrosis initiators RIP1 and RIP3 [79]. The induction of apoptosis by increasing the LIP is presumably due to increased oxidative stress leading to mitochondrial damage [80,81,82]. However, it has been seen in breast cancer cells that depletion of iron is also capable of inducing apoptosis through p53 induction [83]. This may suggest that increased availability of iron is another mechanism of evading apoptosis. Recently, ferroptosis has been used to describe an iron-mediated cell death in non-apoptotic cells [78,84]. Ferroptosis, first described in 2012, is characterized by reactive oxygen species (ROS)-induced, iron-mediated lipid peroxidation [85]. To characterize ferroptosis as an independent form of iron-mediated cell death, it has been shown that it can be inhibited by iron chelation [85] and the GPx-4 inhibitor, liproxstatin-1 [86]. However, both apoptosis and necroptosis inhibitors had no effect on this mode of cell death [85].

Taken together, it is clear that iron utilization is essential for many dyregulated cellular functions that are considered to be hallmarks of cancer including cellular proliferation, DNA repair, and avoidance of apoptosis.

### 3.2. Iron Influx/Efflux and Cancer

Canonically, iron is made more available in cancer cells because of changes in both its uptake and efflux. TfRs are frequently overexpressed in a variety of cancers including breast, brain, lung, liver, ovarian, and prostate cancers, thereby allowing for the increased uptake of iron [87]. TfR expression can actually be used to predict breast cancer progression with a hazard ratio 3.54 [64]. While TfRs are predominantly regulated post-transcriptionally by the IRP/IRE system, they are also regulated at the transcriptional level. The collection of transcription factors regulating TfR have oncogenic implications (reviewed in detail in [87]).

A major contributor to TfR upregulation in cancer cells is the classic oncogene c-Myc [87]. Using a chromatin immunoprecipitation approach, O’Donnell et al. showed that c-Myc binds directly to an E-box motif (CACGTG) in intron 1 of the TfR gene. Both siRNA knockdown and inhibition of TfR in c-Myc responsive B-cell lymphoma cells resulted in G1 arrest through the activation of p21 and p53 along with the induction of p53 mediated apoptosis [88,89]. It has been shown in C57MG breast cancer cells that TfR and Myc are target genes in the Wnt pathway [90].

In addition to c-Myc, TfRs are also transcriptionally upregulated in response to hypoxia. Increased iron influx protects against hypoxia because iron is an essential cofactor for the delivery of oxygen to cells. Expression of Hypoxia Inducible Factor-1α (HIF-1α) under hypoxic conditions stimulates both TfR and erythropoietin (EPO) [91]. This allows for increased iron uptake and erythropoiesis for efficient delivery of oxygen. HIF-1α promotes iron uptake by increasing TfR expression [92]. The TfR promoter region contains a hypoxia response element (HRE) that binds to HIF-1α and enhances expression. For example, HeLa and K562 cells exposed to 16 h of hypoxia have a 2- to 3-fold increase in TfR expression [93]. With tumor hypoxia being common in solid tumors, HIF-1α is often constituently expressed. HIF-1α expression is linked to: angiogenesis, tumor metastasis, and poor cancer outcomes [94]. Therefore, constitutive expression of HIF-1α allows for the overexpression of TfR linking the preferential uptake of iron to disease progression.

As tumors preferentially increase TfR expression, increased expression of proteins that facilitate endosomal internalization of the di-ferric Tf-TfR complex allow for increased iron uptake. Increased expression of the various subunits that make up vacuolar-ATPases (V-ATPases) have been implicated in numerous cancers including brain, breast, liver, pancreatic, ovarian, and GI cancers [83,95]. V-ATPases are localized to the membrane of secretory vesicles (i.e., endosomes); they hydrolyze ATP in order to pump protons into the vesicle, which lowers the pH, facilitating the dissociation the di-ferric Tf-TfR complex—iron is released [95]. A recent study by Schneider et al. has shown that V-ATPase inhibition results in an inhibition of di-ferric Tf-TfR internalization, iron depletion, G1/S phase arrest, p53 induction, and apoptosis [83]. Within the endosome, six-transmembrane epithelial antigens of the prostate family members (STEAP) 2, 3, and 4 act as ferrireductases allowing for the release of Fe^2+^ into the cytosol [96,97,98]. The STEAP family of proteins are overexpressed in a wide variety of cancers [18,97]. Overexpression of STEAP3 aids in maintaining colorectal cancer cell growth in vitro and tumor growth in vivo under hypo-iron conditions [99].

**Figure 2:**
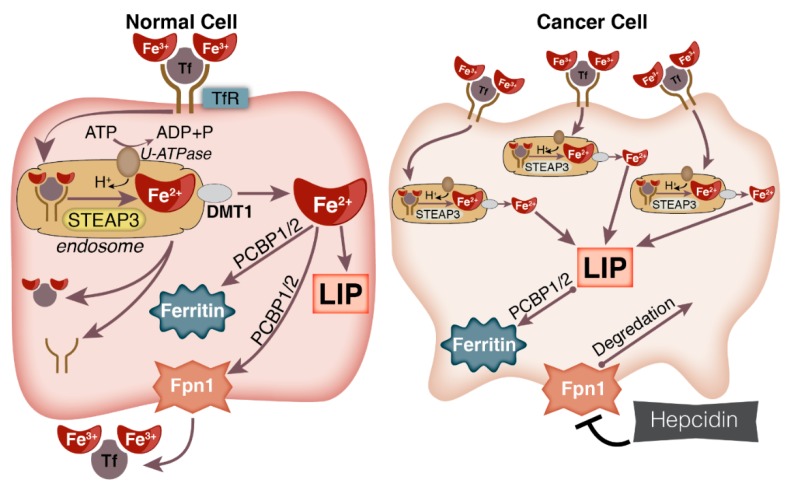
Alterations in iron uptake and efflux in cancer cells compared to normal cells. Cancer cells often exhibit increased expression of TfR that can increase iron uptake as well as increased hepcidin leading to degradation of FPN-1, thereby limiting export of iron from the cell. To facilitate increased uptake of iron, cancer cells have also been shown to increase expression of V-ATPase and STEAP3, which are important for the endosomal internalization of the di-ferric Tf-TfR complex and facilitation of the release of ferrous iron into the cytosol.

To maintain an increased LIP, cancer cells also limit their ability to export iron. Hepcidin is a hormone secreted from the liver that controls systemic iron levels by binding to FPN-1 on enterocytes, reducing iron export into the circulation [22]. When in the circulation, hepcidin will bind to FPN-1 on the basolateral surface of the cell resulting in internalization and degradation of the complex [100]. The result of this degradation is limited export of iron from the cell. In a variety of cancers, hepcidin expression is frequently increased, reviewed in [101]. A study in pancreatic cancer patients showed that low expression of FPN-1 in tumors coupled with high expression of hepcidin predicts poor survival outcomes [102]. In breast cancer tissues, FPN-1 expression was significantly decreased and was indicative of poorly-differentiated tissue. When FPN-1 was overexpressed in breast cancer cells, there was a marked reduction in tumor growth in mouse mammary fat pads. In a cohort of >800 women analyzed for expression of FPN-1 and hepcidin in breast cancer cells independent of other breast cancer markers, high Fpn-1/low hepcidin showed a 10-year survival of over 90% while those with low FPN-1/high hepcidin expression showed a 10-year survival as low as 43% [103]. In the prostate cancer cell lines, LNCap, DU145, and PC3, knock-down of hepcidin via siRNA resulted in significant decreases in proliferative capacity [104]. Additionally, the iron export chaperone, PCBP-1, has tumor suppressive capabilities in prostate cancer cells [28]. However, this effect was shown as a result of alterations in the MAPK1 pathway and not directly linked to iron export. Therefore, evidence suggests cancer cells preferentially reduce their capacity to export iron. This, coupled with the increased iron uptake, allows for an increased LIP (Figure 2).

### 3.3. Mitochondrial Iron Metabolism and Cancer

Mitochondria act as the central hub for utilization of iron as they house both the heme synthesis and Fe-S biogenesis machinery [43]. While there is increasing cytosolic iron in neoplasms, it is unclear if this results in an increase in the mitochondrial LIP [105]. Among the 16 genes identified in the IRGS predictive of breast cancer progression, mitochondrial proteins involved included ISCU, MFRN-1 (SLC25A37), SFXN1, SFXN5 [64]. Each of these proteins is involved in Fe-S biogenesis. Increases in the ubiquitous mitochondrial iron importer, MFRN-2, have been seen in head and neck cancer suggesting increased mitochondrial uptake of iron to complement increased uptake of cellular iron [106]. Overexpression of MtFt resulted in reductions in heme synthesis capabilities, altered IRP/IRE binding, and tumor growth inhibition [107,108].

Increasing uptake of mitochondrial iron is likely necessary to meet the increased demand for iron needed for increase rates of proliferation along with other cellular survival functions. An important protein involved in these functions may be frataxin. Frataxin is a protein that can have reduced expression due to genetic mutation, as in the neurodegenerative disease, Fredrich’s Ataxia. Frataxin homozygous knockout mouse models were embryonically lethal [109]; reduction of frataxin caused accumulation of mitochondrial iron and increased oxidative damage [110]. Frataxin is upregulated in glioblastoma tumors and participates in the hypoxia-induced stress response of tumors. Thus, frataxin may function in an antioxidant capacity to enhance tumor survival and progression by sequestering excess iron, allowing for its utilization rather accumulation that could lead to increased oxidative distress [111]. However, this idea is paradoxical to the canonical understanding of frataxin’s role in cancer. Frataxin has previously been shown to function as a tumor suppressor as its overexpression in a colon cancer model has been shown to decrease growth rates, inhibit soft agar colony formation, and prevent tumor growth in mice [112]. It has been shown that p53 directly regulates frataxin expression as Pifithrin-α inhibition of p53 expression resulted in a decrease of frataxin expression. This study showed that frataxin is transcriptionally regulated by p53 through a proximal promoter region containing a p53 response element [113]. Frataxin, despite being poorly understood, is an example of an iron utilization protein that has a complex but important role in carcinogenesis.

A new development in attempts to understand and target biogenesis of mitochondrial Fe-S centers in cancer are NEET proteins. NEET proteins are involved in trafficking Fe-S clusters to the cytosol. This class of proteins has been thoroughly reviewed in Mintler et al. [47]. MitoNEET overexpression in triple negative breast cancer cells, MDA-MB-231, resulted in enhanced tumor xenograft growth, resistance to nutrient starvation induced autophagy, and an increase in oxidative phosphorylation proteins [114]. Suppressing NAF-1 and MitoNEET reduced breast cancer tumor growth by up to 90% by increasing mitochondrial iron, cellular ROS, and enhancing autophagy [115]. NAF-1 loss also activates apoptosis in breast cancer [116]. NAF-1 and MitoNEET overexpression in breast cancer, hepatocellular carcinoma, pancreatic cancer, cervical cancer [47,117,118,119] are linked to tumor progression.

In addition to increased Fe-S biogenesis and trafficking, heme synthesis is also altered in cancer. The rate-limiting enzyme of heme synthesis is ALAS. ALAS has been shown in non-small cell lung cancer (NSCLC) to be upregulated promoting cellular proliferation [120]. It has been shown in NSCLC cells that introducing heme-sequestering peptides suppresses tumor growth in vivo [121]. Along with ALAS, NSCLC cells also have higher levels of heme synthesis proteins and increased rates of heme synthesis [122]. PPIX has been shown to accumulate in non-proliferating, dormant, metastatic prostate cancer cells as a result of FECH downregulation [123,124]. These results further suggest that increased heme synthesis may support tumor growth.

### 3.4. Mitochondrial Dysfunction, ROS, and Iron Storage in Carcinogenesis

A novel model of cancer development has emerged as the “Horse and Cart” theory of carcinogenesis that ties together metabolic and genetic theories of cancer [125,126]. This model proposes that genetic instability associated with the induction of cancer may be driven by perturbations in mitochondrial oxidative metabolism leading to increased steady-state levels of ROS and disruption in redox sensitive signaling processes governing growth and development. Dysfunctional mitochondrial oxidative metabolism often leads to a “build-up” of electrons at sites capable of mediating one-electron reductions of O_2_, leading to an increase in the steady-state levels of intracellular ROS. The increased steady-state levels of ROS are proposed to drive increased genomic instability, inability to differentiate, loss of control of cell proliferation, immortalization, and the progression to the malignant state [125,126,127,128,129]. The Warburg effect also explains the increased glucose uptake of tumor tissue relative to adjacent normal tissues [130]. However, the increased glucose uptake may be for utilization in the pentose phosphate pathway to generate reducing equivalents, i.e., NADPH. Increased NADPH may help mitigate increases of ROS in cancer cells due to increased steady-state levels of superoxide (O_2_^•−^) and hydrogen peroxide (H_2_O_2_) by driving the detoxification of hydroperoxides via the glutathione peroxidases and peroxiredoxins [131,132].

The increased steady-state levels of O_2_^•−^ and H_2_O_2_ in cancer cells may also have a direct impact on the size of the LIP, thereby impacting both Fe^2+^-O_2_ and Fenton chemistry. This can initiate redox signaling that can contribute to the dysregulation seen in cancer metabolism and biology. Labile iron is readily able to undergo one-electron redox reactions allowing for a feed-forward cascade of oxidations impacting the structure and function of many critical biomolecules. The thermodynamics of some redox reactions involving Fe^3+^/Fe^2+^ are summarized in Table 1 [133].

In the presence of increased H_2_O_2_, Fe^2+^-containing complexes are readily able to participate in Fenton chemistry as it is thermodynamically favorable for biologically relevant complexes to be oxidized by H_2_O_2_ [133]. For example, the one-electron oxidation of Fe^2+^-citrate by H_2_O_2_ has a ΔE^o’^ > 0 (Fe^2+^-citrate/Fe^3+^-citrate, E^o’^ = −100 mV; H_2_O_2_, H^+^/H_2_O, HO^•^, E^o’^ = 320 mV, thus ΔE^o’^ = +220 mV). This indicates that it can be readily oxidized, i.e., the participation of Fe^2+^-citrate in the Fenton reaction is thermodynamically favorable. Other Fe^2+^ complexes are also ready participants, including Fe^2+^-EDTA (ΔE^o’^ = +200 mV) and Fe^2+^-ADP (ΔE^o’^ = +220 mV).

In addition to the ability of Fe^2+^ complexes to react with O_2_ and H_2_O_2_, the reduction of the resulting Fe^3+^-complexes back to Fe^2+^-complexes is of great importance as this redox-cycling of iron will amplify the oxidative damage. This is readily done by cellular reductants, such as ascorbate (vitamin C) [134]. The size of the LIP can be increased by both O_2_^•−^ and H_2_O_2_. For example, the [4Fe-4S] cluster of active, resting-state aconitase has two irons as Fe^2+^ and two as Fe^3+^. The cluster can be oxidized by O_2_^•−^/HO_2_^•^ as well as H_2_O_2_, leaving one Fe^2+^ and three Fe^3+^. The remaining Fe^2+^ dissociates from the cluster as an addition to the LIP, leaving the protein with a [3Fe-4S] cluster; the protein function changes from aconitase to iron response protein-1 (IRP-1). The iron of ferritin can also be mobilized by superoxide. Ferritin is typically thought to control LIP levels. However, under circumstances with increasing O_2_^•−^ and H_2_O_2_, iron stored in ferritin may contribute to the LIP. This labilization allows for Fe^2+^ to participate in both Fe^2+^-O_2_ and Fenton chemistry leading to iron mediated oxidative damage to DNA, proteins, lipid membranes (i.e., ferroptosis), etc.

Ferritin’s contributions to tumorigenesis are not well understood. Ferritin light chain (Ft-L) is upregulated in glioblastoma tumors relative to low-grade gliomas and knockdown of Ft-L results in inhibition of glioma cell growth [135]. In glioblastoma stem-like cells, ferritin was upregulated and was a shown to be a negative prognostic indicator. Inhibition of ferritin in these cells resulted in decreased cell growth both in vivo and in vitro [136]. In metastatic melanoma, Ft-L was upregulated in an autologous lymph node metastases compared to primary tumors [137]. Ft-L knockdown in lymph node metastatic melanoma cells resulted in an inhibition of cell growth and chemo-invasion increasing the sensitivity to oxidative stress and the induction of apoptosis. Ft-H has also been implicated in breast cancer progression [138]. Ft-H mRNA levels in tumor samples collected from 42 patients revealed a significant correlation between Ft-H and axillary lymph node status, presence of metastatic disease, and clinical state (stage I/II vs. stage III/IV). Ft-H has also been shown to correlate with proliferative activity in luminal and basal subtypes of breast cancer [20]. The MCF-7 cell line (luminal subtype B), MDA-MB-231 (basal subtype), and MDA-MB-468 cell lines (basal subtype) have a high proliferative capacity and contain the highest LIPs when compared to other breast cancer cell lines (T47D, MCF-10A, and 184 A1 cell lines [20]. Ft-H mRNA expression showed a strong correlation with ki-67, superoxide dismutase (SOD) activity, ROS, and LIP measures. These observations support Ft as a contributor of iron to the LIP; the iron in Ft can be labilized through reduction of the Fe^3+^-complexes in ferritin under conditions of increased ROS.

Ferritin may also act in an anti-oxidant capacity as it is transcriptionally regulated by the anti-oxidant transcription factor, Nuclear factor (erythroid-derived 2)-like 2, NRF-2 [139]. NRF-2 is often expressed transiently in response to acute oxidant stresses. However, constitutive NRF-2 expression is often implicated in cancer cell resistance, which results in constitutive upregulation of ferritin When constituently overexpressed, NRF-2/Ft has been shown to act in an oncogenic signaling capacity [136]. This oncogenic switch may occur through the activation of the pro-mitotic Forkhead Box M1 transcription factor (FoxM1). The induction of FoxM1 by ferritin also may contribute to cancer progression through de-differentiation of tumor cells. However, there is limited understanding regarding the upregulation of FoxM1 by ferritin, which may be indirect through redox signaling. FoxM1 has been shown in cancer cells to induce epithelial to mesenchymal transitions (EMT) and promotes cancer cell stemness [140,141,142,143]. Increasing the expression of ferritin may contribute to the LIP and increased cellular oxidation under more constitutively oxidized conditions, as in a cancer cell.

**Figure 3:**
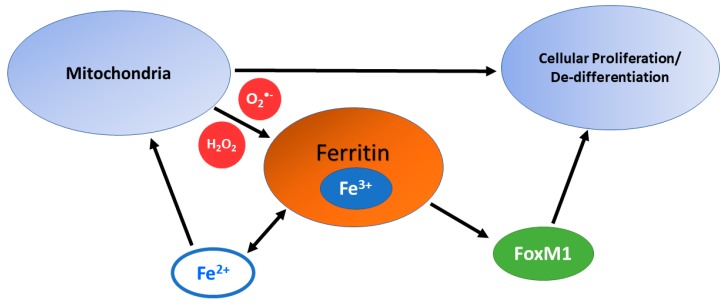
Potential consequences from overexpression of ferritin in cancer cells. Ferritin holds iron that can be labilized by increased levels of ROS generated by mitochondria. This “liberated” iron could be taken up by mitochondria to support proliferation. At the same time, ferritin could act in a signaling capacity by increasing expression of the promitotic, oncogenic transcription factor, FoxM1.

Due to the contributions that labile iron makes to ROS generation from Fe^2+^-O_2_ and Fenton chemistry, sequestering iron should exhibit an anti-oxidant effect and promote cell cycle arrest. Despite its canonical role as an intracellular iron chelator, in the context of increased O_2_^•−^ levels (among other ROS) as is commonly the case in tumor cells, iron stored in ferritin may contribute to the LIP. This would further increase the amount of iron available for the tumor to utilize. Therefore, in both a thermodynamic and cell signaling context, it may be more favorable to have iron stored as a Fe^3+^-complex in ferritin for disease progression. Increased levels of ROS would allow for the tumor cells to more readily access the iron being stored while simultaneously preventing further genomic instability from general Fenton chemistry (Figure 3).

## 4. Targeting Iron in Cancer

Because of the apparent dependence on iron for the survival of cancer cells, iron metabolism has been suggested as a therapeutic target. The targeting of iron in cancer has traditionally been done through chelation therapy. The iron chelator desferrioxamine (DFO), was initially used for the treatment of β-thalassemia associated iron overload. In 1988, DFO was shown to have anti-tumor potential in patient-derived neuroblastoma cells [144]. In these neuroblastoma cells, DFO was shown to have cytotoxic effects along with the promotion of growth inhibition. DFO has since been shown to promote apoptosis and exhibit antiproliferative effects in cervical cancer cells. This effect was also shown by a separate iron chelator, Deferiprone [145]. In leukemia cells, DFO has been shown to reduce proliferative capacity and inhibit DNA synthesis [146]. DFO has been shown in ovarian cancer cells to induce a G0/G1, S-phase block as well as inducing apoptosis [147].

From these in vitro experiments iron chelation has shown to be a promising method of targeting iron metabolism. However, there have been mixed results using DFO clinically. In 1990, a phase II trial in 9 neuroblastoma patients treated with DFO resulted in a response from 7 of 9 patients [148]. One patient had a 48% reduction in tumor volume. A clinical trial of 57 neuroblastoma patients treated with DFO, cyclophosphamide, etoposide, carboplatin, and thiotepa showed 26 complete responses, 24 partial responses, 3 minor responses, and 4 patients progressed on trial [149]. In 2011, a study of 10 patients with advanced hepatocellular carcinoma treated with DFO showed a 20% partial response rate [150]. One patient with a large hepatocellular lesion had a complete response. However, despite the positive results of DFO, a 1994 study revealed that DFO was unable to produce a complete or partial response in 10 pediatric neuroblastoma patients. In this trial, DFO also showed significant toxicities including blurred vision, dizziness, and leg cramping [151]. Due to confounding results regarding DFO efficacy, a new generation of iron chelators is being evaluated clinically including Deferiprone and Deferasirox. Both Deferasirox and Deferiprone have been shown to exhibit anti-cancer effects in triple negative breast cancer cells and prostate cancer cells, respectively [152,153].

Converse to chelation therapy is ascorbate therapy. High dose ascorbate, IV delivery, has been shown to be an efficacious anti-cancer agent when used as an adjuvant in numerous cancer therapies. It has been shown that ascorbate’s mechanism of action is in part through the labilization of iron. Ascorbate is capable of reducing and labilizing iron from proteins resulting in an increased LIP capable of generating detrimental ROS through Fe^2+^-O_2_ reactions and Fenton chemistry [21,154]. Ascorbate has been shown to selectively increase the labile iron pool in non-small cell lung cancer and glioblastoma cells relative to normal cells in vitro. The labilization of iron by ascorbate is central to its cytotoxic effects. The selective toxicity of ascorbate was mitigated in vitro by an siRNA knockdown of TfR and overexpression of ferritin [21].

Pharmacological levels of ascorbate (mM concentrations in plasma) had been shown to be safe and efficacious in terminally ill cancer patients in the 1970s [155,156,157,158]. However, a randomized, double blinded clinical trial of orally delivered ascorbate showed no therapeutic benefit when compared to a placebo control [159]. These results blunted interest in high dose ascorbate as a novel cancer therapy until a pharmacokinetic study revealed that plasma concentrations of ascorbate saturate at ≈220 μM for doses delivered orally [160]. Since the determination that plasma concentrations reaching pharmacological levels cannot be achieved orally, several clinical trials have been completed. A small study in 2004 demonstrated that the use of intravenous, pharmacological ascorbate to be safe and well tolerated in renal cell carcinoma, colorectal cancer, pancreatic cancer, non-Hodgkin’s lymphoma, and breast cancer [161]. In 2012, a phase I clinical trial of 14 patients was completed combining pharmacological ascorbate with gemcitabine and erlotinib for patients with stage IV pancreatic ductal carcinoma. Of the 14 patients, 9 completed the trial. Following completion of the 8 week trial, 7 patients had stable disease and 2 showed progression (per RESIST criteria) [162]. A 2014 phase I clinical trial of 14 stage IV pancreas cancer patients combining pharmacological ascorbate with gemcitabine showed potential efficacy for pharmacological ascorbate. Patients receiving pharmacological ascorbate with gemcitabine had an average overall survival of 15 ± 2 months and average time to progression of 26 ± 7 weeks. These results were a great improvement compared to historical averages of 6 month survival and 9 week time to progression [163]. Pharmacological ascorbate has also shown potential for the treatment of glioblastoma tumors. In a phase I trial of 11 glioblastoma patients treated with pharmacological ascorbate, temozolomide, and radiation the average progression-free survival was shown to be 13.3 months with an average overall survival was 21.5 months in MGMT expressing tumors [21]. This was an improvement on the historical median progression-free survival of 9.4 months and overall survival of 18.2 months [164]. These results suggest that the targeting of iron metabolism within a tumor through increased labilization of iron and oxidative stress is a potentially novel alternative to chelation therapy.

## 5. Conclusions

A growing body of data suggest that iron dysregulation plays an integral role in the development of neoplastic disease. Canonically, the metabolic dysregulation of iron could contribute to unifying the concepts embodied in both the genetic and metabolic theories of cancer. There is a large amount of data showing that tumors preferentially upregulate iron import pathways (i.e., TfR upregulation via c-Myc, EGFR, etc.) while preventing its export (i.e., upregulation of hepcidin resulting in FPN-1 degradation). The increased uptake of iron into both the cytosolic and mitochondrial compartments of cells may be contributing to increased utilization of Fe (e.g., Mfn-2/ISCU/Frataxin upregulation) and ultimately increased cellular proliferation (i.e., cyclinD1/CDK4 overexpression—p21 down regulation). The concept of tumors having increased levels of LIP is consistent with the novel “Horse and Cart” model of impaired mitochondrial function leading to carcinogenesis and cancer progression [125,126]. With increased levels of O_2_^•−^ and H_2_O_2_, the labilization of iron is a thermodynamically favorable process that allows for iron levels to remain high with detrimental oxidations and inappropriate signaling enhanced by Fe^3+^/Fe^2+^ redox recycling. In this regard, the metabolic dysregulation of iron and the nature of the LIP appear to link both metabolic and genetic theories of cancer. Continued research into the complex interactions of intracellular iron metabolism in cancer versus normal cells may provide novel, fundamental biochemical insights into the understanding of cancer biology leading to advances in cancer therapy. This has the potential to allow for context dependent, targeted therapies for improving patient outcomes.

## Figures and Tables

**Table 1:** Standard one-electron reduction potentials involving iron complexes at pH = 7.0 [133].

Couple	E^o’^ (mV)
Fe^3+^-transferrin/ Fe^2+^-transferrin	−400 (pH = 7.3)
Fe^3+^-ferritin/ Fe^2+^-ferritin	−190
O_2_/ O_2_^•−^	−160 (−330) ^a^
Fe^3+^-DETAPAC/ Fe^2+^-DETAPAC	30
Fe^3+^-citrate/ Fe^2+^-citrate	100
Fe^3+^-ADP/ Fe^2+^-ADP	100
Fe^3+^/Fe^2+^ (aqueous)	110
Fe^3+^-EDTA/Fe^2+^-EDTA	120
H_2_O_2_, H^+^/H_2_O, HO^•^	320 mV

^a^ −160 mM if using 1 M O_2_ or −330 mV if using 1 atm O_2_ for the standard state.
